# Does Living Near a Superfund Site Contribute to Higher Polychlorinated Biphenyl (PCB) Exposure?

**DOI:** 10.1289/ehp.8827

**Published:** 2006-01-23

**Authors:** Anna L. Choi, Jonathan I. Levy, Douglas W. Dockery, Louise M. Ryan, Paige E. Tolbert, Larisa M. Altshul, Susan A. Korrick

**Affiliations:** 1 Department of Environmental Health and; 2 Department of Biostatistics, Harvard School of Public Health, Boston, Massachusetts, USA; 3 Department of Environmental and Occupational Health, Rollins School of Public Health, Emory University, Atlanta, Georgia, USA; 4 Channing Laboratory, Department of Medicine, Brigham and Women’s Hospital, Harvard Medical School, Boston, Massachusetts, USA

**Keywords:** exposure pathways, geographic information systems, hazardous waste site, newborn, PCBs, polychlorinated biphenyls, remediation, Superfund

## Abstract

We assessed determinants of cord serum polychlorinated biphenyl (PCB) levels among 720 infants born between 1993 and 1998 to mothers living near a PCB-contaminated Superfund site in Massachusetts, measuring the sum of 51 PCB congeners (∑PCB) and ascertaining maternal address, diet, sociodemographics, and exposure risk factors. Addresses were geocoded to obtain distance to the Superfund site and neighborhood characteristics. We modeled log_10_(∑PCB) as a function of potential individual and neighborhood risk factors, mapping model residuals to assess spatial correlates of PCB exposure. Similar analyses were performed for light (mono–tetra) and heavy (penta–deca) PCBs to assess potential differences in exposure pathways as a function of relative volatility. PCB-118 (relatively prevalent in site sediments and cord serum) was assessed separately. The geometric mean of ∑PCB levels was 0.40 (range, 0.068–18.14) ng/g serum. Maternal age and birthplace were the strongest predictors of ∑PCB levels. Maternal consumption of organ meat and local dairy products was associated with higher and smoking and previous lactation with lower ∑PCB levels. Infants born later in the study had lower ∑PCB levels, likely due to temporal declines in exposure and site remediation in 1994–1995. No association was found between ∑PCB levels and residential distance from the Superfund site. Similar results were found with light and heavy PCBs and PCB-118. Previously reported demographic (age) and other (lactation, smoking, diet) correlates of PCB exposure, as well as local factors (consumption of local dairy products and Superfund site dredging) but not residential proximity to the site, were important determinants of cord serum PCB levels in the study community.

Polychlorinated biphenyls (PCBs) are persistent synthetic organic chemical pollutants found in air, water, sediments, and soil. Because of concern over their toxicity and persistence in the environment, the manufacture of PCBs was banned in the United States in 1977, resulting in declines in environmental PCB levels (Longnecker et al. 1997). However, exposure to PCBs continues because of their presence in products manufactured before 1977, the disposal of PCB-contaminated products in landfills and hazardous waste sites, and their environmental persistence and bioaccumulative characteristics.

The developing fetus is particularly vulnerable to exposure to environmental toxins (Fein et al. 1983). PCBs readily cross the placenta, and prenatal PCB exposure has been associated with decreased birth weight (Fein et al. 1984; Patandin et al. 1998) and decrements in cognitive function in childhood (Jacobson et al. 1996; Stewart et al. 2003; Vreugdenhil et al. 2002). However, some studies have not demonstrated adverse associations of early-life PCB exposures with prenatal growth or childhood cognition (Gladen and Rogan 1991; Gray et al. 2005). Given their potential health hazards, it is important to understand risk factors (including potentially remediable ones) for PCB exposure among infants and children.

Among general population samples, diet, particularly consumption of contaminated fish and other animal products, is a major source of PCB exposure. Other potential pathways for PCB exposure include inhalation and dermal contact, both occupationally and in the ambient environment (DeCaprio et al. 2005; Löffler and Bavel 2000). Among reproductive-age women, reported correlates of serum PCB levels include older age, alcohol consumption, parity, and lactation (Jacobson et al. 1984; Rogan et al. 1986). However, risk factors for nonoccupational PCB exposure vary among populations and regions. Residential proximity to a contaminated site may be an important risk factor for PCB exposure, as it may capture both direct exposure pathways (inhalation or dermal contact) and socioeconomic- or lifestyle-related exposure risks.

The New Bedford Harbor in southeastern Massachusetts is contaminated with PCBs as a result of waste disposal from local industry from the 1940s until 1977. The most PCB-contaminated sediment or “hot spot” was in the harbor estuary adjacent to a capacitor manufacturer (Figure 1) (Weaver 1984). In 1982 the harbor was designated a Superfund site. As part of the remediation plan, the most contaminated sediments were dredged between April 1994 and September 1995 [U.S. Environmental Protection Agency (U.S. EPA) 1999].

The present study was undertaken to assess whether residential proximity to this PCB-contaminated site or related local factors (e.g., consumption of locally produced foods) were associated with higher cord serum PCB levels among infants of mothers living near the harbor. We characterized PCB exposure incorporating known exposure pathways and individual risk factors with geographic information system methods to assess spatial correlates of cord serum PCB levels. We evaluated exposure pathways as well as temporal variability in exposure for PCBs and, because of their unique toxicologic properties, dioxin toxic equivalent (TEQ) levels for dioxin-like PCBs (Van den Berg et al. 1998).

## Materials and Methods

### Study population

Study participants were part of an ongoing cohort study of PCBs and child development. Mother–infant pairs were recruited just after birth at St. Luke’s Hospital in New Bedford, Massachusetts, between March 1993 and December 1998. Dredging of PCB-contaminated New Bedford Harbor sediments occurred in the middle of the study recruitment period (April 1994 to September 1995). Participation was limited to consenting mothers (18 or more years of age) who had resided in one of the four towns (New Bedford, Acushnet, Fairhaven, Dartmouth) bordering New Bedford Harbor for the duration of their pregnancy. Infants born by cesarean section were excluded. Infants who required high-grade neonatal care or were otherwise not available for a newborn examination were not included in the study.

Of the 788 participants in the birth cohort study, 37 did not have a cord serum sample (not collected at birth or lost during laboratory sample preparation). We excluded one infant whose mother’s address was missing, two younger twins, and 28 younger siblings, leaving a total of 720 infants available for analysis.

The human subjects committees of Harvard School of Public Health, Brigham and Women’s Hospital of Boston, and St. Luke’s Hospital of New Bedford, Massachusetts, approved the protocol of this study. Data were collected after written informed consents were obtained from study mothers.

### Cord blood PCB levels

Cord blood samples were obtained at birth in Vacutainer tubes and centrifuged, and the serum fraction was removed. The serum was stored in solvent-rinsed glass vials with Teflon-lined caps at –20°C until extraction. Analyses were performed by the Harvard School of Public Health Organic Chemistry Laboratory. Cord blood analytic methods and quality control procedures are described elsewhere (Korrick et al. 2000). Briefly, 51 individual PCB congeners were measured using liquid–liquid extraction and extract analysis by capillary column gas chromatography with electron capture detection. Confirmatory analyses were done with microelectron capture detection and a capillary column of different polarity. Serum lipids were not measured because of insufficient sample volume. PCB concentrations were reported as the sum of 51 congeners (∑PCB) in units of nanograms of analyte per gram of serum. We also grouped PCB levels into light PCBs (sum of 14 mono- to tetrachlorinated biphenyls) and heavy PCBs (sum of 37 pentato decachlorinated biphenyls) according to their elution order and relative volatility (Cullen et al. 1996). These two groups were chosen *a priori* based on the hypothesis that PCB exposure pathways may vary by their relative volatility.

The 51 congeners were chosen based on their toxicity, persistence in the environment or human samples, and presence in New Bedford environmental samples; these included a subset of mono-*ortho* dioxin-like PCBs (congeners 105, 118, 156, 167, and 189). The dioxin TEQ concentration for the dioxin-like PCBs was calculated (Van den Berg et al. 1998) and expressed in parts per trillion (ppt) lipid, assuming 0.17% lipid for cord serum based on our laboratory’s data and published values (Altshul LM, personal communication; Denkins et al. 2000). PCB-118 was chosen a priori for individual assessment. It was prevalent in harbor sediments consistent with the predominant Aroclors used by the area’s industries (Brown and Wagner 1990; Weaver 1984). In addition, it was disproportionately prevalent in our serum samples; cord serum levels of PCB-118 were comparable with levels observed in other population-based surveys (Korrick et al. 2000) despite overall PCB levels being substantially lower than most other populations (Longnecker et al. 2003).

### Dietary assessment

Mothers completed a semiquantitative food-frequency questionnaire during a home evaluation of the child at age 2 weeks. The mothers reported diet histories before and during pregnancy. Twenty-four items from the food-frequency questionnaire were collapsed into six groups: meat (including organ meat), poultry, dairy, eggs, grains, and fish. We further considered fish in four subcategories: tuna; dark-meat fish (mackerel, blue fish, salmon, sardines, and swordfish); other fish (including catfish), and shellfish. In addition, mother’s self-reported consumption of locally grown produce, dairy products (including eggs), meat (including chicken), fish, game, and wine were determined as binary (yes/no) variables.

### Occupation, gardening, and other potentially PCB-exposure–related activities

Mothers’ potential occupational PCB exposure (including working with paints, sealants, caulking compounds, and lubricants), gardening, and other potentially PCB-exposure–related activities (including use of pesticides and fertilizers) were determined by interviewer-administered questionnaire at the 2-week evaluation. Total person-years of exposure were calculated separately for occupation, gardening, and other. Potential exposures were reported as the sum of years from self-reports of engagement in these activities for at least 1 day per week. For each exposure pathway, we divided person-years of potential exposure into three categories: zero and below and above the 75th percentile of nonzero values.

### Other risk factors

We determined maternal age, birthplace, race, education, marital status, reproductive history, pregnancy smoking and alcohol consumption, residential history, household income, and infant’s race and sex from the 2-week questionnaire and maternal and infant medical records.

### Geographic information systems

Home address for the duration of the mother’s pregnancy was geocoded by Mapping Analytics (Rochester, NY), a commercial geocoding firm previously shown to have good (96%) accuracy (Krieger et al. 2001). We used the geocoded residence location for mapping, calculating distance from the Superfund site, and retrieving Census block group data.

A map of New Bedford Harbor PCB levels (U.S. EPA 2001) was aligned to the Massachusetts town boundaries (MassGIS 2002; scale 1:25,000 meter units) using ArcGIS (ESRI Inc., Redlands, CA) to estimate the latitude and longitude of the harbor hot spot. Residential distance (in miles) from the hot spot was used as an index of potential site-related PCB exposure.

Indoor PCB sources include pre-1977 sealants, electrical appliances, and light fixtures (Balfanz et al. 1993; Vorhees et al. 1997). We did not have information about individual home characteristics, but as a proxy, we calculated the fraction of houses built between 1940 and 1979 compared with the total number of houses built through 1990, using 1990 Census block group data.

We constructed neighborhood socioeconomic indices based on 1990 Census block group data (Krieger et al. 2003): *a*) crowding—percentage of households with more than one person per room; *b*) poverty—percentage of persons below the federally defined poverty line ($12,647 for a family of four in 1989); *c*) low income—percentage of households with income less than 60% of the U.S. median household income ($18,000); *d*) median household income; *e*) high education—percentage of persons, 25 or more years of age, with at least 4 years of college; and *f* ) low education—percentage of persons, 25 or more years of age, with less than a 12th grade education.

### Statistical analysis

The cord serum PCB levels were highly positively skewed and were log_10_ transformed for linear regression analyses. Univariate and bivariate associations were explored. Associations between log PCB levels and continuous covariates were assessed using scatter plot smoothing (Venables and Ripley 1997) to examine any nonlinear relationships.

Potential exposure risk factors were divided into those associated with exposure pathways—dietary, inhalation, and dermal exposure sources—and those related to individual characteristics. A set of core individual characteristics was included in each exposure pathway analysis: maternal age and birthplace, smoking during pregnancy, previous lactation, child’s date of birth and sex, dredging period, and household income. Individual socioeconomic indicators (maternal education and race) were also included in models assessing neighborhood socioeconomic indicators and PCB levels. Multivariate models for log PCB included the core individual characteristics and exposure pathway covariates significant (*p* < 0.10) in at least one of the individual pathway models for at least one of the PCB measures (∑PCB, heavy PCBs, light PCBs and PCB-118). Regression results are reported as the relative (percent) increase in PCB level associated with each predictor, calculated as the antilog of the regression coefficient and 95% confidence intervals.

PCB levels were mapped and a smoothed surface was fitted by kriging (Cressie 1991) using ArcGIS Geostatistical Analyst (ESRI Inc.). We estimated the surface by an inverse-distance weighted average of 25 neighboring points chosen on the basis of a small-prediction mean square error and a reasonable area to detect local spatial variability. We restricted this mapping to residences within a 5-mile radius of the hot spot. Similar mapping was performed for multivariate model residuals to provide information on any unmeasured spatial correlates of PCB exposure. To protect the confidentiality of participants, each residence location was offset by a random amount generated from a normal distribution with mean zero and standard deviation (SD) equal to 1% of the SD of residence latitudes and longitudes.

Generalized additive models (Hastie and Tibshirani 1990) were fit in S-Plus (version 3.4; Insightful Corp., Seattle, WA) to assess temporal variability in PCB levels. The span parameter with the lowest Akaike information criterion (AIC) for each PCB measure was chosen. Linear regression models were fit in SAS (version 8.2; SAS Institute Inc., Cary, NC).

## Results

Cord serum PCB levels had geometric means (SDs) as follows: ∑PCB, 0.40 (2.02) ng/g with a range of 0.068–18.14 ng/g; heavy PCBs, 0.33 (2.09) ng/g with a range of 0.035–11.91 ng/g; light PCBs, 0.063 (2.12) ng/g with a range of 0.0074–6.23 ng/g; PCB-118, 0.035 (2.37) ng/g with a range of 0–2.05 ng/g; and dioxin-like PCB TEQs, 4.40 (2.39) ppt lipid, with a range of 0–151.5 ppt lipid.

Maternal, infant, and household characteristics are shown in Table 1. Twenty percent of mothers were born outside of the United States (14% from Portugal, the Azores, or Cape Verde). Most (58%) had an educational level of high school or less, and 70% had an annual household income of < $40,000; 75% of the study population resided within 3.9 miles of the hot spot. Half of the infants were born after the harbor was dredged (October 1995 and later).

Maternal age at the infant’s birth was strongly associated with cord serum PCB levels, which declined over time, with additional declines after the harbor dredging was completed (Table 1, Figure 2). Mothers who were born in Portugal, the Azores, or Cape Verde and female infants had significantly higher cord serum PCB levels (Table 1). After adjustment for maternal age, prior lactation and higher household income were associated with lower cord serum PCB levels. Maternal smoking during pregnancy was also associated with lower PCB levels (Table 1). These parameters were defined as core covariates and included in subsequent analyses. Maternal marital status, alcohol consumption during pregnancy, and infant race were not associated with serum PCB levels.

PCB associations with maternal diet before and during pregnancy were essentially the same. We report the results of analyses assessing diet during pregnancy. Maternal intake of organ meats (liver, tripe, kidney, bone marrow) was significantly associated with higher PCB levels (*p* < 0.05) for ∑PCB, light PCBs, and PCB-118 after adjustment for the base model covariates (*p* = 0.05 for heavy PCBs) (Table 2). Consumption of local dairy products (including eggs) was associated with significantly higher levels of ∑PCB and heavy PCBs. Consumption of dark fish was positively associated with PCB levels, but this association was only marginally significant for light PCBs and PCB-118 (Table 2).

Mothers who were long-term gardeners had infants with lower heavy PCB and PCB-118 levels than infants of mothers who did not garden; however, this association was based on a very small sample size (*n* = 9) and therefore was not included in our final multivariate model. Otherwise, we found no consistent association of cord serum PCB levels with PCB-related occupations or activities, distance of residence from the hot spot, or age of homes in the child’s neighborhood (Table 3). Although there was a tendency for infants born to mothers living in poor or low-income neighborhoods to have higher light PCB levels than those born to mothers living in other neighborhoods, these associations were not significant (Table 4).

We constructed multivariate models including core covariates and significant covariates from the pathway analyses (Table 5). Maternal age and birthplace (in Portugal, the Azores, or Cape Verde) remained the strongest predictors of cord serum PCB levels (*p* < 0.001). In addition, infants born late in the study had significantly lower PCB levels than infants born early in the study (Table 5). Even with adjustment for infant birth date, infants born after dredging had significantly lower light PCB and PCB-118 levels, with near significance for ∑PCB levels (Table 5). Covariate-adjusted smoothed plots of ∑PCB, heavy PCB, light PCB, and PCB-118 levels by infant date of birth corroborate the apparent independent dredging effect (Figure 2). Mother’s prior lactation and smoking during pregnancy were significantly associated with lower PCB levels, and maternal consumption of organ meat and locally produced dairy were associated significantly with higher PCB levels (Table 5).

**Figure 1:**
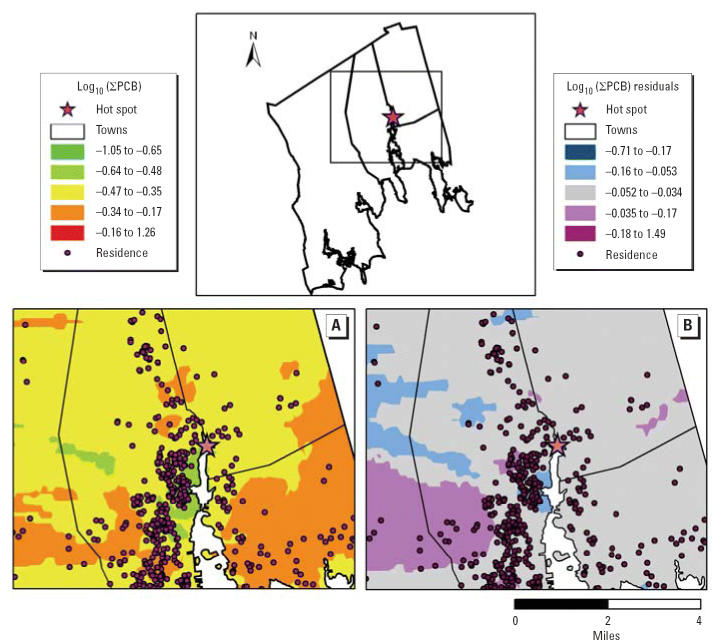
Spatial distribution of log_10_(∑PCB) levels (*A*) and residuals from the multivariate model (*B*) restricting data to a 5-mile neighborhood of the hot spot. Residence locations are jittered with 1% random noise to protect confidentiality of participants.

Maps of unadjusted log_10_(∑PCB) levels (Figure 1A) and log_10_(∑PCB) residuals from the multivariate adjusted model (Figure 1B) showed spatial variability in PCB levels but no relationship to proximity of residence to the PCB hot spot. Similar results were found with heavy and light PCB levels.

Results of pathway analyses for dioxin-like PCB TEQs were similar to those of the four other PCB measures. Significant predictors of higher PCB TEQ concentrations included older maternal age; maternal birth in Portugal, the Azores, or Cape Verde; and consumption of red meat during pregnancy. Mother’s previous lactation, smoking during pregnancy, and infant birth at end of the study were associated with lower PCB TEQs.

## Discussion

We found no evidence that living closer to the New Bedford Harbor Superfund site was associated with increased cord serum PCB levels either in the crude unadjusted means or after adjusting for other risk factors for PCB exposure in the study population.

**Figure 2:**
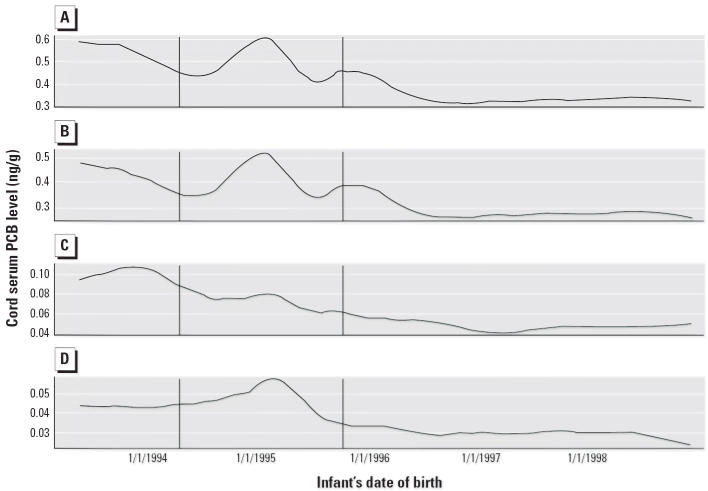
Covariate-adjusted smoothed plots of predicted ∑PCB (*A*), heavy PCB (*B*), light PCB (*C*), and PCB-118 (*D*) levels versus infant’s date of birth. Vertical lines denote the start and stop dates for dredging of contaminated New Bedford Harbor sediments. Plots are adjusted for child’s sex, maternal age, birthplace, smoking during pregnancy, previous lactation, household income, and diet (consumption of organ meat, red meat, local dairy, and dark fish).

However, children born before or during dredging had consistently higher cord serum PCB levels than children born after dredging, even after we accounted for birth date (Table 5, Figure 2), suggesting a possible effect of the PCB-contaminated site and its dredging on cord blood PCB levels. Serum levels of light PCBs were more strongly associated with dredging than were heavy PCBs (Table 5). This finding suggests that differences in PCB volatility affect exposure risks potentially associated with the site. Furthermore, for PCB-118, a dioxin-like pentachlorinated biphenyl disproportionately prevalent in study samples, the dredging effect was more significant than the temporal decline, with near-constant concentrations before dredging, an increase during dredging, and a significant decline after dredging (Figure 2). Overall, these results support modest, transient increases in cord serum PCB levels during dredging, with significant declines in serum PCB levels observed after dredging, particularly for the more volatile PCBs and PCB-118 (Table 5, Figure 2). The apparent differential effects of remediation on cord serum levels of various congeners are notable given possible congener-specific differences in toxicity.

In addition to the previously described dredging associations, maternal consumption of locally produced dairy products—an exposure risk factor potentially related to the contaminated site—was associated with higher cord serum PCB levels (Tables 2, 5).

The most important predictor of elevated cord serum PCB levels was older maternal age at the birth of the study infant. Older age is a well-established risk factor for increased serum organochlorine concentrations, presumably as a consequence of cumulative exposure and temporal trends in exposure (Kutz et al. 1991). In multivariate models, we found that older maternal age and earlier birth year were both associated with elevated cord blood PCB levels, indicating both cumulative exposure and temporal trend effects.

Mothers born in Portugal, the Azores, or Cape Verde had infants with substantially higher cord serum PCB levels than mothers born in the United States, Canada, or other countries, even after adjustment for diet or other lifestyle covariates that may vary by country of origin (Table 5). Although this observed association may be a chance finding or consequent to residual confounding by diet or lifestyle, it is also consistent with potentially higher early-life exposure to PCBs resulting in higher serum levels during pregnancy. For example, PCB contamination is present in fish species from southern Europe and the Atlantic Ocean along the Azore Islands (Stefanelli et al. 2004). Higher early-life exposure to PCBs has been associated with higher serum levels in adulthood among other populations (Rylander et al. 1997).

We confirmed the previously reported association of prior lactation with lower serum PCB levels, which is likely due to PCB excretion in milk (Fitzgerald et al. 1998; Jensen 1991). Smoking during pregnancy was also associated with lower cord serum PCBs. Previous studies have been inconsistent regarding the association of PCBs with smoking; maternal smoking during pregnancy was associated with higher newborn PCB levels in one study (Lackman et al. 2000) but not in others (Fein et al. 1984; Rogan et al. 1986). Smoking may decrease organochlorine concentrations by enhancing their metabolism via smoking-related induction of cytochrome P450 enzymes (Deutch and Hansen 1999; Zevin and Benowitz 1999). Long-term gardening was associated with lower cord serum PCBs (Table 3), opposite to the hypothesized effect. However, the small number of long-term gardeners (*n* = 9) suggests that chance and/or confounding may explain this finding. Of note, the final multivariate model (Table 5) was unchanged by the addition of gardening (data not shown).

In addition to these correlates of exposure, maternal consumption of organ meat and local dairy products (Tables 2, 5) was associated with significantly higher cord blood PCB levels, but other potential dietary risks (including fish intake) were not. Although fish and animal products have been identified as important sources of general population exposure to PCBs and dioxins in some studies (Laden et al. 1999; Patandin et al. 1999), levels of PCBs and dioxins in fish and other foods have been declining (Hays and Aylward 2003). Contaminated areas of the harbor were closed to fishing in 1979 (Agency for Toxic Substances and Disease Registry 1995), 14 years before we started this study. A lack of association of local fish consumption with serum PCB levels is consistent with the lag between last likely intake of the most contaminated fish and our exposure assessment.

Other evaluated risk factors did not explain heterogeneity in cord serum PCB levels. Although serum concentrations of PCB-exposed workers are higher than those of the general population (Wolff et al. 1982), the small number of mothers with potential occupational exposure limited the statistical power to detect such associations. Furthermore, the age of study mothers was such that most of their occupational (and other) activities occurred after the ban on PCBs.

Neighborhood socioeconomic status and age of housing were not associated with increased cord PCB levels. Although manufacturers incorporated PCBs in building materials and light fixtures during a well-defined time period (Balfanz et al. 1993), house age was not a good predictor of indoor air PCB concentrations in previous studies in New Bedford (Vorhees et al. 1997). This measure does not capture renovations or other potential indoor PCB sources such as electrical appliances or fluorescent lights. Moreover, the neighborhood distribution of home ages is an imperfect proxy for the age of the specific home of interest.

Correlates of cord serum PCBs did not vary much by the different congener groupings assessed. For example, the exposure pathways we observed for the heavy PCBs and ∑PCBs were quite similar (Table 5). This is likely because the correlation of ∑PCB levels with heavy PCBs was much higher (*r* = 0.99) than with light PCBs (*r* = 0.76), consistent with the predominance of heavy PCBs in the sum. Except for diet, correlates of PCB TEQ exposure were also similar to other PCB concentration measures. Specifically, maternal consumption of red meat, but not organ meat, was associated with significantly higher PCB TEQs. Patandin et al. (1999) also found meat to be a major contributor to dietary intake of PCB TEQs. Because the congeners are weighted by dioxin-like activity, these findings provide insights into the correlates of potential toxicity, about which very little is known.

There are several limitations in the interpretation of our findings. First, the median serum PCB level in our cohort was about one-quarter of the overall median in a recent review of 10 studies of PCBs and neurodevelopment (Longnecker et al. 2003). Despite this limitation, our findings corroborate previously established correlates of serum PCB levels, including age and secular trends. In addition, the use of simplified proxies for some exposure pathways limited our ability to determine the relative contribution of various routes of exposure to cord serum PCB levels. In particular, it could be argued that residential distance from the site does not capture outdoor concentrations because it ignores prevailing winds. However, the maps of cord serum PCB levels and model residuals do not indicate any likely wind-related spatial patterns with this region’s prevailing wind direction from the south-southwest (Cullen et al. 1996). In addition, it is possible that including household income and other demographic variables reduced our ability to characterize exposure pathways by overadjusting for these indirect correlates of exposure. However, sensitivity analyses demonstrated that this was not the case. For example, results of our pathway analyses were not substantially changed by removing income from the model. Lastly, the cross-sectional nature of this analysis limits the certainty of inferences regarding the observed temporal-and dredging-associated differences in serum PCB levels.

In conclusion, our findings among New Bedford area infants suggest that maternal residence near a Superfund site per se does not lead to higher cord serum PCB levels independent of other exposure risk factors, such as maternal age, birthplace, diet, previous lactation, pregnancy smoking, and infant date of birth. However, there was evidence of an important local impact on exposure risk as shown by increased cord serum PCB levels in association with maternal local dairy consumption and lower cord serum PCB levels after site dredging.

## Figures and Tables

**Table 1:** Maternal, infant, and household characteristics and associated PCB levels (unadjusted geometric means, ng/g serum) among 720 mother–infant pairs in the Greater New Bedford area.

	*n* (%)	∑PCB	Heavy PCBs	Light PCBs	PCB-118
Total	720	0.40	0.33	0.063	0.035
Maternal age (years)					
< 20	104 (15)	0.31	0.23	0.061	0.026
20–24	224 (31)	0.30	0.24	0.054	0.027
25–29	204 (28)	0.42	0.35	0.061	0.040
30–34	138 (19)	0.58	0.49	0.079	0.048
≥ 35	50 (7)	0.73	0.63	0.083	0.051
*p*-Trend		*p* < 0.0001	*p* < 0.0001	*p* < 0.0001	*p* < 0.0001
Sex of infant					
Male	370 (51)	0.38	0.31	0.062	0.033
Female	350 (49)	0.42	0.34	0.065	0.037
*p*-Value		*p* = 0.07	*p* = 0.06	*p* = 0.35	*p* = 0.11
Infant’s date of birth					
Before dredging	140 (19)	0.53	0.41	0.100	0.038
During dredging	216 (30)	0.46	0.38	0.071	0.045
After dredging	364 (51)	0.33	0.27	0.049	0.029
*p*-Heterogeneity		*p* < 0.0001	*p* < 0.0001	*p* < 0.0001	*p* < 0.0001
Maternal race					
Black/African American	24 (4)	0.33	0.27	0.059	0.027
Latino	46 (8)	0.37	0.30	0.063	0.037
Other^a^	52 (9)	0.48	0.39	0.077	0.045
Non-Hispanic white	460 (79)	0.40	0.33	0.060	0.035
*p*-Heterogeneity		*p* = 0.13	*p* = 0.13	*p* = 0.13	*p* = 0.10
Maternal birthplace					
Other countries	33 (6)	0.49	0.40	0.077	0.048
Portugal/Azores/Cape Verde	80 (14)	0.59	0.48	0.090	0.063
United States/Canada	464 (80)	0.37	0.30	0.057	0.032
*p*-Heterogeneity		*p* < 0.0001	*p* < 0.0001	*p* < 0.0001	*p* < 0.0001
Maternal education					
High school or less	335 (58)	0.38	0.31	0.061	0.035
Some college or higher	247 (42)	0.42	0.34	0.062	0.037
*p*-Value		*p* = 0.18	*p* = 0.14	*p* = 0.71	*p* = 0.36
Previous lactation					
0–6 months	514 (88)	0.39	0.32	0.061	0.036
> 6 months	68 (12)	0.44	0.37	0.060	0.035
*p*-Value		*p* = 0.19	*p* = 0.14	*p* = 0.86	*p* = 0.91
Maternal smoking during pregnancy					
No	392 (67)	0.43	0.35	0.032	0.042
Yes	190 (33)	0.34	0.27	0.054	0.026
*p*-Value		*p* < 0.0001	*p* < 0.0001	*p* = 0.002	*p* < 0.0001
Annual household income					
< $40,000	383 (70)	0.39	0.32	0.061	0.035
≥ $40,000	164 (30)	0.46	0.38	0.066	0.042
*p*-Value		*p* = 0.01	*p* = 0.01	*p* = 0.27	*p* = 0.02

a Other race includes Asian and Native American and nonwhite Cape Verdean.

**Table 2:** Percent change^a^ in newborn cord serum PCB levels associated with maternal dietary exposure.^b^

	No. with given dietary exposure^c^	∑PCB	Heavy PCBs	Light PCBs	PCB-118
General diet					
Red meat (> 2/week)	394	7	6	13*	13
Organ meat (> 1/month)	49	23**	20*	32**	31**
Chicken/turkey (> 1/month)	511	2	4	–1	11
Dairy (> 5/day)	257	–5	–6	–3	–4
Eggs (> 2/week)	296	–6	–5	–5	–4
Dark fish (> 1/month)	120	10	9	15*	15*
Other fish (> 2/week)	112	1	2	–3	5
Tuna (> 2/week)	197	–1	–2	1	–1
Shellfish (> 2/week)	249	–1	–1	–4	–1
Grain (> 3/day)	264	–3	–3	–2	0
Locally grown food (yes)					
Fish	54	6	8	2	8
Wine	27	6	7	8	16
Produce	358	–6	–7	–5	–4
Dairy	53	27**	31**	9	23*
Game	20	–3	–4	–3	–6
Meat	25	–16	–21	–4	–17

a Reflects percent change in a multiplicative scale, obtained by exponentiating the regression coefficient in the log-transformed PCB model.

b Adjusted for base variables (maternal age, birthplace, smoking during pregnancy, previous lactation, household income, child’s date of birth and sex, and dredging period).

c Represents number of individuals in each food consumption group from a total sample of 531 with nonmissing diet data.

* *p* < 0.10;

** *p* < 0.05.

**Table 3:** Percent change^a^ in newborn cord serum PCB levels associated with proxies for maternal dermal and/or inhalation exposure.^b^

	*n*	∑PCB	Heavy PCBs	Light PCBs	PCB-118
Occupation					
None	407	Reference	Reference	Reference	Reference
≤ 10 years	102	–4	–3	–2	–3
> 10 years	33	17	16	18	17
Gardening					
None	505	Reference	Reference	Reference	Reference
≤ 10 years	27	–3	–5	2	11
> 10 years	9	–26	–34**	–14	–48**
Other activities					
None	458	Reference	Reference	Reference	Reference
≤ 10 years	58	6	7	5	13
> 10 years	25	–10	–9	–13	–4
Residence distance from hot spot (miles)					
0.2–1.5	134	Reference	Reference	Reference	Reference
1.6–2.8	140	2	2	7	2
2.9–3.8	133	4	4	4	3
3.9–10.8	135	7	5	15*	5
Neighborhood houses built between 1940–1979 (%)					
0–20	123	Reference	Reference	Reference	Reference
21–32	143	2	0	1	–3
33–60	158	–1	0	–5	3
61–100	118	2	2	0	2

a Reflects percent change in a multiplicative scale, obtained by exponentiating the regression coefficient in the log-transformed PCB model.

b Adjusted for base variables (maternal age, birthplace, smoking during pregnancy, previous lactation, household income, child’s date of birth and sex, and dredging period).

* *p* < 0.10;

** *p* < 0.05.

**Table 4:** Percent change^a^ in newborn cord serum PCB levels associated with quartiles of household neighborhood characteristics.^b^

Characteristics	∑PCB	Heavy PCBs	Light PCBs	PCB-118
Crowding (%)				
0	Reference	Reference	Reference	Reference
> 0–2	–8	–9	–7	–8
3–4	–2	–2	–3	–9
5–16	1	–1	10*	1
*p*-Trend	*p* = 0.78	*p* = 0.94	*p* = 0.19	*p* = 0.70
Poverty (%)				
0–5	Reference	Reference	Reference	Reference
6–11	0	1	–6	3
12–25	–1	–3	1	–1
26–55	4	–1	15	6
*p*-Trend	*p* = 0.69	*p* = 0.95	*p* = 0.11	*p* = 0.37
Low income (%)				
0–32	Reference	Reference	Reference	Reference
33–42	–8	–8	–12	–9
43–54	7	5	9	2
55–89	3	0	6	2
*p*-Trend	*p* = 0.32	*p* = 0.54	*p* = 0.13	*p* = 0.20
Median household income ($)				
7,000–17,000	Reference	Reference	Reference	Reference
17,001–25,000	12	14	7	–2
25,001–30,000	–4	–2	–12	–9
30,001–68,000	–1	3	–7	–10%
*p*-Trend	*p* = 0.57	*p* = 0.90	*p* = 0.24	*p* = 0.37
Low education—less than high school (%)				
0–33	Reference	Reference	Reference	Reference
34–48	–4	–5	–4	1
49–65	4	1	11	10
66–80	–6	–9	2	–1
*p*-Trend	*p* = 0.71	*p* = 0.45	*p* = 0.42	*p* = 0.79
College education (%)				
0–4	Reference	Reference	Reference	Reference
5–9	–4	–1	–11	–1
10–13	–3	0	–10	0
14–47	–9*	–5	–17**	–7%
*p*-Trend	*p* = 0.70	*p* = 0.95	*p* = 0.37	*p* = 0.97

a Reflects percent change in a multiplicative scale, obtained by exponentiating the regression coefficient in the log-transformed PCB model.

b Adjusted for base variables (maternal age, birthplace, smoking during pregnancy, previous lactation, household income, child’s date of birth and sex, and dredging period), maternal education, and race.

* *p* < 0.10;

** *p* < 0.05.

**Table 5:** Percent change^a^ (95% confidence interval) in newborn cord serum PCB levels as a function of significant maternal and infant predictors (*p* < 0.10).

	∑PCB (*n* = 541, *R*^2^ = 38%)	Heavy PCBs (*n* = 541, *R*^2^ = 37%)	Light PCBs (*n* = 541, *R*^2^ = 28%)	PCB-118 (*n* = 541, *R*^2^ = 30%)
Child characteristics				
Male sex	–7 (–15 to 3)	–7 (–16 to 2)	–1 (–11 to 10)	–8 (–19 to 4)
Date of birth (years)^b^	–36 (–52 to –13)#	–31 (–50 to –6)**	–43 (–60 to –21)#	–13 (–41 to 27)
Child born before/during dredging	17 (–3 to 40)*	16 (–4 to 41)	26 (2 to 55)**	39 (10 to 75)#
Mother’s demographics				
Maternal age (years)^b^	36 (29 to 43)#	40 (33 to 47)#	17 (11 to 24)#	25 (17 to 33)#
Born outside United States/Canada				
Portugal/Azores/Cape Verde	42 (23 to 63)#	41 (21 to 63)#	42 (21 to 67)#	69 (42 to 102)#
Other countries	20 (–3 to 48)*	20 (–4 to 50)	20 (–6 to 54)	33 (1 to 74)**
Previous lactation (> 6months)	–25 (–35 to –12)#	–25 (–36 to –12)#	–26 (–38 to –12)#	–33 (–45 to 19)#
Smoking during pregnancy (yes)	–11 (–20 to –1)**	–12 (–21 to –2)**	–11 (–21 to 1)*	–29 (–38 to –19)#
Household income (≥ $40000)	1 (–13 to 10)	–4 (–15 to 9)	5 (–8 to 20)	4 (–10 to 21)
Mother’s diet				
Organ meat (> 1/month)	21 (2 to 44)**	19 (–1 to 42)*	30 (7 to 59)#	30 (5 to 62)**
Local dairy (yes)	19 (1 to 39)**	19 (1 to 41)**	8 (–10 to 29)	16 (–6 to 42)
Red meat (> 2/week)	7 (–5 to 19)	5 (–6 to 18)	12 (–1 to 27)*	12 (–2 to 29)
Dark fish (> 1/month)	8 (–4 to 21)	7 (–6 to 21)	13 (–1 to 30)*	14 (–2 to 33)*

a Reflects percent change in a multiplicative scale, obtained by exponentiating the regression coefficient in the log-transformed PCB model.

b Effect estimates per 5 years.

* *p* < 0.10;

** *p* < 0.05;

# *p* < 0.01.
